# Curcumin and its Potential for Systemic Targeting of Inflamm-Aging and Metabolic Reprogramming in Cancer

**DOI:** 10.3390/ijms20051180

**Published:** 2019-03-08

**Authors:** Renata Novak Kujundžić, Višnja Stepanić, Lidija Milković, Ana Čipak Gašparović, Marko Tomljanović, Koraljka Gall Trošelj

**Affiliations:** 1 Laboratory for Epigenomics, Ruđer Bošković Institute, Division of Molecular Medicine, 10 000 Zagreb, Croatia; rnovak@irb.hr (R.N.K.); stepanic@irb.hr (V.S.); Marko.Tomljanovic@irb.hr (M.T.); 2 Laboratory for Oxidative Stress (LabOS), Ruđer Bošković Institute, Division of Molecular Medicine, 10 000 Zagreb, Croatia; Lidija.Milkovic@irb.hr (L.M.); Ana.Cipak.Gasparovic@irb.hr (A.Č.G.)

**Keywords:** curcumin, oxidative metabolites, inflamm-aging, cancer, metabolic reprogramming, direct protein binding, IL-17, STAT3, SHMT2

## Abstract

Pleiotropic effects of curcumin have been the subject of intensive research. The interest in this molecule for preventive medicine may further increase because of its potential to modulate inflamm-aging. Although direct data related to its effect on inflamm-aging does not exist, there is a strong possibility that its well-known anti-inflammatory properties may be relevant to this phenomenon. Curcumin’s binding to various proteins, which was shown to be dependent on cellular oxidative status, is yet another feature for exploration in depth. Finally, the binding of curcumin to various metabolic enzymes is crucial to curcumin’s interference with powerful metabolic machinery, and can also be crucial for metabolic reprogramming of cancer cells. This review offers a synthesis and functional links that may better explain older data, some observational, in light of the most recent findings on curcumin. Our focus is on its modes of action that have the potential to alleviate specific morbidities of the 21st century.

## 1. Introduction

During the past one and a half decades, we have been witnessing increased interest in natural compounds and their applications to everyday life. This fact should not be surprising, especially in the field of preventive medicine. Thanks to various interventions including, but not limited to, vaccination, high hygienic standards, avoidance of smoking and alcohol abuse, healthy diet, maintenance of body weight and exercising, human life span is extended. According to the World Health Organization (WHO), the average global life expectancy for those born in 2016 is 72.0 years (males 69.8 years; females 74.2 years), with a significant differential depending on the geographical region: African region (only) 61.2 years, versus European region 77.5 years [1]. The United Nations Department of Economic and Social Affairs (DESA) has forecasted that global life expectancy at birth, for both sexes combined, is going to rise to 76.9 years by years 2045–2050 [2].

However, an inevitable trade-off of extended lifespan is the increased incidence of various age-related diseases, of which cancer is certainly a very important one.

Chronic inflammation is common to aging and age-related diseases. The concept of a causal relationship between inflammation and cancer dates back to Rudolf Virchow who suggested, in his lectures in 1858, that the “lymphoreticular infiltrate” reflects the origin of cancer at sites of chronic inflammation. He pointed out that there are striking similarities between ulcers, wound healing, and cancer [3,4]. More than a century later, Harold F. Dvorak published an essay in the New England Journal of Medicine entitled “Tumors: Wounds that do not heal. Similarities between tumor stroma generation and wound-healing.” He proposed that solid tumors act as parasites that promote a wound-healing response to acquire the stroma needed for their survival and growth [5]. Aging is often accompanied by an impaired healing response [6], accumulation of senescent cells, and chronic inflammation [7].

The link between aging and inflammation is very accurately coined in the term “inflamm-aging”, which denotes the up-regulation of certain pro-inflammatory cytokines at older ages, and is associated with chronic diseases. This term was originally introduced in 2006 [8], to describe the imbalance between inflammatory and anti-inflammatory networks, which results in a low grade chronic, age-associated, pro-inflammatory status. Currently, deregulated cytokine production is appreciated as a very important consequence of the remodeling of the immune system in old age (recently reviewed) [9]. It has been recognized that, in older subjects, high levels of interleukins IL-6 and IL-1, tumor necrosis factor-alpha (TNF-α), and C-reactive protein (CRP) are associated with an increased risk of morbidity and mortality [10].

The rate of cancer incidence is increasing, especially in the Western world. According to GLOBOCAN 2018, there have been 18.1 million new reported cases of cancer in 2018 (9.5 million males and 8.6 million females). The same database predicted 9.6 million cancer deaths in 2018 (5.4 million males and 4.2 million females) [11]. For 2040, the International Agency for Research on Cancer predicted 29.5 million newly diagnosed cancer patients, and 16.4 million cancer deaths [12].

Early detection screenings represent a valid effort in fighting cancer. In the same setting, the era of biotechnology implemented in molecular medicine, enables an insightful understanding of the molecular mechanisms of action of natural compounds which have been used in traditional medicine for centuries. One such compound is curcumin, which possesses properties relevant to successful cancer chemoprevention. This is particularly important for the elder population.

## 2. Curcumin and Inflamm-aging

The natural source of curcumin is the rhizome of the medicinal plant, *Curcuma longa*, a perennial herb in the family *Zingiberaceae* [13]. The curcuminoid complex, found in the rhizome of turmeric (2.5–6%) contains: curcumin (CUR - diferuloylmethane, ~85%); demethoxycurcumin (DEM, ~15%); bis-demethoxycurcumin (bis-DEM, ~5%) and cyclocurcumin [14]. In the commercially available formulations, the major curcuminoid complexes are reported to be in a similar range: 77% of CUR; 17% of DEM; and 3% of bis-DEM) [15]. By 2022, the U.S. curcumin market is expected to approach 40 million dollars [16]. It is also recognized that over 52% of curcumin production will be used for pharmaceutical applications, in the U.S.

Well-known for its healing properties, curcumin has been extensively used in traditional Ayurveda, Unani and Siddha medicine for treating various diseases. An early mention of curcumin in modern medical literature was in 1937, appearing in the Lancet, one of the most prestigious clinical medical journals [17]. The article, describing curcumin applications to humans, was written by Albert Oppenheimer—then an assistant professor at the American University of Beirut, Lebanon, who applied curcumin orally (up to 800 mg daily) for the treatment of 67 patients suffering from various forms of subacute, recurrent, or chronic cholecystitis. The positive therapeutic response recorded then, was the basis for future interest in curcumin and its healing properties, especially its anti-inflammatory properties, which were among the first studied [18].

Is it reasonable to conclude that the anti-inflammatory property of curcumin may be used for alleviating inflamm-aging? If that is so, can it explain the beneficial effects of curcumin in various experimental models of diseases of elderly humans? We will present some older and some very recent data which we consider central in the context of this puzzle.

In2009, Smith et al. published data showing that the total projected cancer incidence will increase in the U.S. by approximately 45%, in only 20 years, from 1.6 million (2010) to 2.3 million, in 2030 [19]. This prediction seems to be very accurate since, for 2019, 1,762,450 new cases and 606,880 cancer deaths were estimated to occur in the U.S. [20]. The Smith’s study predicted something that was way ahead of the time when the article was published. A 67% increase in cancer incidence was anticipated for older adults [19]. Eight years later, in 2017, Nolen et al. have shown that cancer prevalence and cancer incidence increases until ages 85-89, after which the rates decrease until 100+ [21].

Inflammation has been recognized as a strong contributor to the acquisition of core hallmark cancer capabilities [22]. The most recent data convincingly show that the high level of pro-inflammatory cytokines (such as IL-6 and IL-8) may predict some crucial prognostic parameters in cancer patients [23,24]. In addition to an increased level of IL-6 and IL-8, colorectal cancer patients also have alterations in their serum amino-acid profile. Low levels of serum glutamine, histidine, alanine and high glycine levels were shown to be associated with advanced stage of cancer and with poor cancer-specific survival (*N* = 336; univariate analysis) [25].

There are also quite convincing data on neuroinflammation as a crucial process in the pathogenesis of the two the most common neurodegenerative diseases in the elder population: Alzheimer’s disease (AD) and Parkinson’s disease (PD) [26]. Based on numerous data, it has been proposed that both disorders are strongly related to inflamm-aging [27,28]. As recently reviewed, three shared strong mediators of inflammatory reaction are increased in patients suffering from these two disorders: IL-6, IL-1β and TNF-α [29].

These inflammatory molecules are integrative parts of the canonical activation of the NF-kappa B (nuclear factor kappa-light-chain-enhancer of activated B cells; NF-κB) signaling pathway. Generally, this pathway has been considered as protumorigenic, although there are several models showing its opposite action [30]. During the last few years, the interleukin 17 (IL-17) has become the focus of various types of research, including the research related to cancer, Alzheimer’s and Parkinson’s disease. There are several models in which the communication between NF-κB and IL-17 takes the place. The most recent data indicate that IL-17 can promote the proliferation and migration of glioma cells via PI3K/AKT1/NF-κB-p65 activation [31].

### 2.1. Inflamm-Aging, Interleukin-17 and Curcumin

Originally, in 2002, human colonic subepithelial myofibroblasts (SEMFs) were used as a model system for showing that SEMFs secrete IL-6, IL-8, and MCP-1 (Monocyte Chemoattractant Protein-1), in response to IL-17 [32]. The most recent data point out the critical role for IL-17-producing T lymphocytes in sporadic PD, in humans. In vitro, the midbrain neurons (MBNs) were shown to increasingly die consequentially to the upregulation of IL-17 receptor (IL-17R) and NF-κB activation [33]. The involvement of IL-17-producing T lymphocytes in the onset of Alzheimer’s disease was also shown, in the animal model [34].

Although majority studies performed so far indicate the presence/an increase of IL-17 as a negative prognostic factor for cancer patients, the results are not entirely conclusive and depend on numerous factors. These include the type of the tumor, the types of genetic aberrations, which may significantly vary, and the host’s immune response. Of importance, different cell types expressing IL-17 can play different roles, not only in the tumor microenvironment, but, it seems, may provide insight into the overall picture of the malignant tumor. In a cohort of 573 gastric cancer patients [35], high levels of IL-17+ neutrophils were shown in the tumorous tissue, where the high level of IL-17 induced the migration of neutrophils into gastric cancer, via cancer cell–derived CXC chemokines. These neutrophils were further shown to stimulate the proangiogenic activity of tumor cells, both in vitro and in vivo. The increase in IL-17-expressing cells in peripheral blood, particularly Th17, was associated with tumor progression in HNC (Head and Neck Carcinoma) patients (*N* = 120) [36].

While there are many data on curcumin and NF-κB, there are only a few of them in relation to curcumin and its effect on IL-17producing cells. In relation to HNC, it is quite interesting that the testing in 2015 of a novel microgranular curcumin formulation (15 patients and eight healthy volunteers) showed that curcumin strongly decreases the serum level of IL-17 at one hour post- ingestion; *p* = 0.0342 [37].

### 2.2. Breast Tissue Inflamm-Aging and Curcumin

There are many research papers discussing curcumin’s action on sex-hormone related cancer (breast, ovary, prostate), in vitro. In breast cancer cells, the effects obtained depend not only on the status of estrogen and progesterone receptors (estrogen receptor; ER and progesterone receptor; PR), but also on the specific genetic/epigenetic cellular background. A 20 year old paper presented the strong inhibitory action of curcumin alone and in combination with isoflavonoids in both ER-positive human breast cancer cells (MCF-7 and T47D) and ER-negative MDA-MB-231 cells exposed to environmental estrogens (pesticide o,p’-DDT and the pollutants 4-nonylphenol and 4-octylphenol) [38]. The mechanism of curcumin’s inhibitory effect on MCF-7 and MDA-MB-231 was explained four years later [39]. Curcumin exerts antiproliferative effects on MCF-7 cells through: (a) inhibiting the exogenous 17-β estradiol’s stimulatory growth effect on these cells; (b) blocking the expression of downstream ER-responsive genes, pS2 and TGF-α, when applied in high concentrations. In MDA-MB-231 cells, this mechanism was absent. Instead, curcumin strongly decreased the level of MMP-2 (matrix metalloproteinase 2).

In this paper, we reinforce the fact that breast cancer has been recognized as a systemic disease [40]. There is strong experimental data showing that postmenopausal breast cancer in obese women also can be considered as „breast inflamm-aging“ disease. At the outset of the pathophysiological process, subclinical, local breast inflammation, characterized by crown-like structures (CLS) consisting of dead adipocytes surrounded by macrophages, occurs in breast white adipose tissue [41]. This type of inflammation is paralleled by increased NF-κB binding activity joined with elevated levels of proinflammatory mediators and a key enzyme in the biosynthesis of estrogen in menopause, aromatase (estrogen synthase) [42]. Through aromatization of androgen precursors in adipose tissue (through tissue specific reactions), this enzyme makes a strong link between inflammation, obesity, and an increased risk for hormone receptor-positive breast cancer [43].

On the other hand, lipolysis, which is also increased in obesity, results in increased concentrations of free fatty acids [44]. Saturated fatty acids trigger the activation of NF-κB in macrophages resulting in increased production of proinflammatory mediators which additionally induce aromatase in preadipocytes [45]. Thus, there is a complete *circulus vitiosus*, associated with increased activity of NF-κB, which can be modified by curcumin.

As shown in vitro, curcumin can significantly suppress the inductive effects of stearic acid–treated macrophages, on aromatase mRNA and aromatase activity in preadipocytes, through suppression of NF-κB and Akt signaling pathways [46]. In the cited paper [46], a mixture of multiple polyphenols (Zyflamend; curcumin included), was given to animals kept on a high-fat-diet. As a result, the increased levels of aromatase mRNA and its activity in the mammary glands were partially suppressed. Although this data constitutes a solid basis for future research on chemoprevention in humans, there is one obvious problem. Measurement of the effect which occurs locally requires a biopsy. This significant obstacle is one, but not the only one, of the major reasons for serious gaps in this area of research [47].

There is no data, to the best of our knowledge showing the organ-specific, local effects and/or systemic effects of curcumin in humans, which would be gender-specific.

### 2.3. Curcumin and the Concept of Network Medicine

The NF-κB signaling pathway is a master regulator of inflammation-associated signaling pathways. Age-related, tissue-specific, brain inflammation mediated by NF-κB, when joined with Nitric Oxide and Reactive Oxygen Species (NOROS), in macrophages has been recognized as a most significant risk factor for developing Alzheimer’s disease [48]. Activation of this pathway has been also recognized in pathogenesis of PD [49]. Thus, if we focus on the pathophysiology of these three diseases (cancer, AD and PD) and their increased incidence in older subjects, we need to take note of some shared general molecular features, of which the “inflamm-age status” represents a very important factor. It may, indeed, be the strongest indicator of perturbations which affect complex intracellular and intercellular networks which communicate at different levels, as suggested by postulates of Network Medicine. This discipline proposes a highly interconnected nature of the interactome and considers that a gene, protein or metabolite can be implicated in several disease modules. Accordingly, at the molecular level, it leads to the conclusion that diseases are not independent of one another [50].

Can this interconnective dependency explain the beneficial effects of multilevel acting pleiotropic curcumin [51] as a molecule that is considered useful for the prevention of these diseases, as recorded in various models/diseases?

First of all, surprisingly, the combined terms “curcumin” and “inflamm-age” appear in only a few research papers in the PubMed database. Secondly, there are only a few studies based on measuring cytokines in cancer-free human subjects who were taking curcumin. Different types of curcumin formulations given, in addition to various doses applied and duration of administration, makes the picture confusing. In a cohort of 72 migraine patients, only the combined daily administration of 2500 mg of ω-3 fatty acids (2 × 1250 mg) and 80 mg nano-curcumin (1 × 80 mg), led to a significant decrease of TNF-α in the serum (*p* = 0.001). Administration of curcumin alone had no significant effects [52]. However, a decrease of TNF-α was recorded (from 103.90 ± 13.29 ng/dL to 92.56 ± 8.70 ng/dL). The question is whether these subtle changes, although not statistically significant when taking into consideration the whole group of subjects, may be significant for the physiology of a particular patient. This would be in accord with the postulates of personalized medicine.

In a study which included 117 subjects suffering from metabolic syndrome, 1 g of curcumin daily and placebo were given to 59 and 58 subjects, respectively, for a period of eight weeks. In this research study, between-group comparison suggested significantly greater reductions in serum concentrations of TNF-α, IL-6, TGF-β (transforming growth factor beta) and MCP-1 in the curcumin versus the placebo group (*p* < 0.001). When adjusted for potential confounders, changes in all parameters (serum glucose and lipids, baseline serum concentration of the cytokines), except IL-6, remained statistically significant [53].

These two examples show that we are far away from firm conclusions when considering the potential beneficial effects of curcumin. The problem relating to the lack of comprehensive data associated with inflammation markers and curcumin application to healthy subjects, was very accurately addressed by Hewlings and Kalman [54], who concluded that measuring potential benefits of curcumin in healthy populations may be challenging because the benefits may not be as immediate and measurable if (bio)markers are normal at the baseline. Thus, for reaching meaningful conclusions, subjects will need to be followed over an extended time period, which would require a high level of participants’ cooperation. But even if the cooperation and stringent follow-up is provided, caution in interpretation is needed.

The necessity of careful interpretation of all this data and the effects obtained must be put in the context of the most recent findings.

## 3. Oxidative Metabolites of Curcumin

The newest data show that the anti-inflammatory effects of curcumin, those mediated through inhibition of NF-κB [55,56], do not depend on the parent molecule, but rather on its oxidized products [57]. It was very convincingly shown that oxidative metabolites of curcumin adduct to and inhibit IKK β (inhibitor of nuclear factor kappa-B kinase subunit beta; known also as IKK2/NFKBIKB). If the cells were pretreated with *N*-acetylcysteine, a biosynthetic precursor of glutathione (GSH), the potency of curcumin was decreased, probably due to GSH-mediated scavenge and inactivation of curcumin-derived electrophiles. Finally, as concluded in the cited article [57], oxidative metabolites of curcumin, which occur in vitro, adduct to cellular proteins. As the authors stated, this may explain the wide range of cellular targets of curcumin identified in vitro. On the other hand, insufficient bioactivation in vivo may underlie the inconclusive data in human studies. This may mean that healthy humans who exhibit a lower level of oxidative stress, may be less likely to experience benefits from the oxidative activation of curcumin. However, the oxidative stress theory of aging, which is based on the hypothesis that age-associated functional losses are due to the accumulation of NOROS-induced damages [58], joined with recent data on age-related inflammation (inflamm-age), may strongly favor the protective effects of curcumin in older age. Thus, before coming to any final conclusion, some molecular events related to curcumin’s pro-oxidative capability, obtained in vitro, need to be addressed.

Recently published data on the anti-tumorigenic effects of curcumin on CML-derived human leukemic cells, in a xenograft model and in vitro culture system [59], confirmed, mostly indirectly, data obtained in experiments with oxidized curcumin metabolites [57]. In the leukemic cells treated with curcumin, the strong cytotoxic effect was joined with the strongest increase of ROS. On the other hand, when GSH was added into the system, the level of ROS was, expectedly, lower, as was the cytotoxic effect. The most probable explanation for these phenomena may be that GSH associated decrease of ROS consequentially leads to a decreased level of oxidized curcumin metabolites. As a consequence, the binding of curcumin to its potential protein-partners decreases. In the leukemic cell model, these protein-partners were shown to be metabolic enzymes, a few of which, like NQO1 (NAD(P)H Quinone Dehydrogenase 1), are well-known targets of the NRF2 (Nuclear Factor (Erythroid-Derived 2)-Like 2) transcription factor.

In addition to NQO-1, some other enzymes discovered as curcumin-binding partners, such as CBR1 (carbonyl reductase 1), GSTP1 (glutathione-S-transferase phi 1), AKR1C1 (aldo-keto reductase family 1 member 1), GLO1 (Glyoxalase I), exert their detoxifying function in the ROS-related metabolic pathway. It is not surprising then that ectopic overexpression of these enzymes led to the same effect as was gained with GSH: decreased level of ROS accompanied by a decrease in the curcumin’s cytotoxic effect.

### Curcumin and ROS-Producing Compounds

When contemplating these in vitro systems, one may ask whether oxidized curcumin metabolites lay in the background of commonly-recorded synergistic effects when applied with cytotoxic drugs, for example – with cisplatin [60]. It has been shown that in cancer cells cisplatin exposure induces a mitochondria-dependent increase in ROS levels [61]. This ROS may be the fuel for occurrence of oxidized curcumin metabolites, which may block the NF-κB signaling pathway (as already described) [57] and, additionally, decrease the activity of detoxifying enzymes through direct binding. This action probably includes balancing many factors, as curcumin (or its oxidative metabolites?) can restore *NRF2* transcription through demethylation of the *NRF2* promoter (first five CpGs positioned between -1086 and -1226) [62]. Then, on the other hand, curcumin uses its oxidized metabolites for binding to at least some protein targets of NRF2 (as is the case of NQO-1, which is considered as a *bona fide* NRF2 target) [63]. This is a good example of the complexity in the field and, as already mentioned, the necessity for multilevel research approach which needs to include as many as possible explorations of various interactions.

In considering the meaning of these recent discoveries, one should remember that, traditionally, various effective combinations of curcumin are, indeed, those which are based on its combination with the ROS-producing compounds. For example, oligomeric proanthocyanidins, which are known to exhibit anticancer properties are, in some studies, shown as ROS generation activators [64]. The effect seems to be cell-type-specific [65]. The combination of proanthocyanidins and curcumin was investigated in several models of colorectal cancer (cell lines, mice xenografts, colorectal cancer patient-derived organoids), where the combined application showed superior anticancer properties. It has been again shown that the expression of some very important genes/proteins remained intact when compounds were applied separately, while combined application induced a strong change in the activity of some genes. For example, a strong decrease of glucose-6-phosphate dehydrogenase (G6PD), a key enzyme in the Pentose Phosphate Pathway (PPP) was discovered [66]. Deficiency of G6PD was recognized to be associated with a lower cancer risk already in 1965 [67]. However, metabolic reprogramming was (officially) recognized as a hallmark of cancer many years later [22]. With respect to curcumin activity, metabolic reprogramming is becoming a hot topic of scientific interest.

## 4. Curcumin, Cancer and Metabolic Reprogramming

“Diffuse, evolutionary and developmental processes that we diagnose as cancer” [68], comprise, through initiation and progression of the disease, dynamic, stepwise changes in fundamental physiological characteristics of cells and tissues. A variety of stresses, either extrinsic (environmental) or intrinsic (genetic, epigenetic, metabolic), induce damage to cellular components that can lead to malignant transformation. Survival of damaged, potentially dangerous cells strongly depends on stress response pathways. One of the main barriers to propagation of cells that are at risk for malignant transformation is the onset of cellular senescence, a phenomenon that closely relates to cellular metabolism.

### 4.1. Cellular Senescence (CS), Hypoxia and Cancer Metabolic Plasticity

Cellular senescence represents stress response which is needed for permanent arrest of division of damaged cells. It is evolutionary well conserved, highly sophisticated and extremely complex. Although CS is of utmost importance in preventing proliferation of cells that are at risk of malignant transformation, the accumulation of senescent cells in aging tissues can compromise normal tissue microenvironment and facilitate cancer progression [69,70]. We are aware that the role of CS in molecular pathophysiology of many age-associated diseases, not only cancer, is multifaceted and can be detrimental.

Senescent cells are not inert. To the contrary, they are metabolically very active, and capable to secrete a plethora of bioactive molecules - pro-inflammatory cytokines and other factors which are able to change tissue structure. In the setting of cancer, they contribute in creating a cancer permissive microenvironment. Most anti-cancer therapeutic modalities promote senescence, which is beneficial for inhibiting tumor cell proliferation. But, at the same time, it is not selective and can induce senescence of adjacent non-tumor cells with consequential local inflammation, occurrence of secondary tumors and cancer relapse.

There are two hypotheses on the protumorigenic role of cellular senescence. According to a cell non-autonomous model, senescent cells secrete a variety of cytokines and factors which modify surrounding cells that have not fully entered senescence [71]. According to cell autonomous model, some senescent cells can bypass senescence and develop stem cell properties [72]. Malignant tumors are composed of a heterogeneous population of cells. This heterogeneity is not only based on genetically distinct subpopulations of tumor cells, but also on their functional heterogeneity.

Cancer is anything but a static system in terms of metabolism. A prominent characteristic of cancer cells is uncontrolled proliferation, which requires nutrients and energy to accommodate their augmented biosynthetic activity. The difference in glucose metabolism between normal and cancer cells was first noted by Otto Warburg [73]. He observed that cancer cells from ascites “ferment” glucose into lactate, even when enough oxygen, needed for supporting mitochondrial oxidative phosphorylation (OXPHOS), is available. Proliferative cancer cells exhibit a high rate of glycolysis, for meeting their high energetic and biosynthetic requirements. Although glycolysis is less efficient in producing ATP per molecule of glucose, high glycolytic flux may provide more cellular ATP from glycolysis than from OXPHOS. This is important for supporting cellular anabolic reactions [74,75,76]. Otto Warburg originally postulated that cancer cells acquire a defect in the mitochondria that disturbs aerobic respiration. We now know that, although mitochondrial function is often disturbed in cancer, cancer cells retain a substantial capacity to produce energy in the mitochondria.

Considering that rapid proliferation of tumor cells is not always adequately accompanied with vascularization of tumor mass, cancer cells are forced to adapt to nutrient- and oxygen-deprived environment. To do so, cancers select metabolically plastic cells, those with the highest capacity for metabolism reprogramming. This metabolic adaptivity supports cellular survival under different types of stress.

Cellular metabolic phenotype changes considerably as malignant tumors progress. In H-RasV12/E1A transformed fibroblasts, an increase in OXPHOS activity precedes the increase of glycolytic rate [77]. The strong mediator of this switch is transcription factor HIF-1α (Hypoxia-Inducible Factor 1α).

Permanent increase of ROS, a by-product of OXPHOS, has a major role in stabilizing HIF-1α and promoting aerobic glycolysis [78,79,80]. The stability of cellular HIF-1α is post-translationally regulated by O_2_−, Fe^2+^- and α-ketoglutarate - dependent hydroxylation of proline residues. Hydroxylation of HIF-1α promotes its ubiquitination and degradation, thereby preventing transcription of its target genes. An elevated level of cellular ROS oxidizes Fe^2+^ to Fe^3+^, abolishes HIF-1α hydroxylation/ubiquitination/degradation. As a consequence, HIF-1α accumulates in the cell [79] where it promotes pseudohypoxic state, permissive of mitochondrial dysfunction.

In Hep G2 hepatocellular carcinoma cells, application of curcumin led to a significant decrease of HIF-1α protein. It also suppressed its transcriptional activity under hypoxia, which resulted in decreased expression of vascular endothelial growth factor (VEGF), known as a major HIF-1 α target [81]. The most recent data point out the strong simultaneous inhibitory effect of polymeric nano-encapsulated curcumin on HIF-1α and REL A (P65; NFKB3), in lung and breast cancer cell lines [82]. Curcumin’s inhibitory effects on gastric cancer in experimental animals were also shown to be dependent not only on HIF-1α/VEGF decrease (shown immunohistochemically), but also on decrease of STAT3 (Signal Transducer and Activator of Transcription 3) transcription factor [83].

What is the role of STAT3 in cellular metabolism, how it relates to inflammation and senescence, and, finally, what kind of influence curcumin may have in relation to this potent transcription factor?

### 4.2. Curcumin, STAT3, and Modes of Survival

Constitutively activated in various malignancies, STAT3 has an important role in inflammation-associated tumorigenesis [84]. It has recently been reported that curcumin inhibits highly active STAT3 in H-Ras transformed breast epithelial cells (H-Ras MCF10A), through direct binding to its cysteine residue 259. This cysteine (Cys) is critical for STAT3 phosphorylation, dimerization, nuclear translocation and DNA binding [85], upon activation by various cytokines, hormones and growth factors.

In addition to inflammation-associated tumorigenesis [86], STAT3 is mandatory for the survival of cells, which, upon bypassing oncogene-induced senescence in premalignant lesions, acquire stem cell properties. This was recently shown in a model of pancreatic cancer [87]. The mechanisms by which cells circumvent senescence in tumors that spontaneously develop from premalignant lesions are still not fully elucidated.

A prominent feature of senescent cells is STAT3 decrease, due to its proteasomal degradation [88]. Considering STAT3 involvement in promoting mitochondrial functions which are needed for cancer cell stemness and metabolic reprogramming, cancer cells that bypass senescence and acquire stem cell properties are sensitive to depletion and/or inactivation of STAT3. Napabucasin, a small molecule compound which was developed to inhibit several pathways in cancer stem cell-like cells, exerts at least some of its therapeutic activity through binding to the SH2 domain of STAT3 and, consequentially, suppresses STAT3 activity [89]. Accordingly, curcumin’s binding to STAT3 Cys259 will result in multiple consequences, in addition to those which were recently discovered [85].

**Figure 1:**
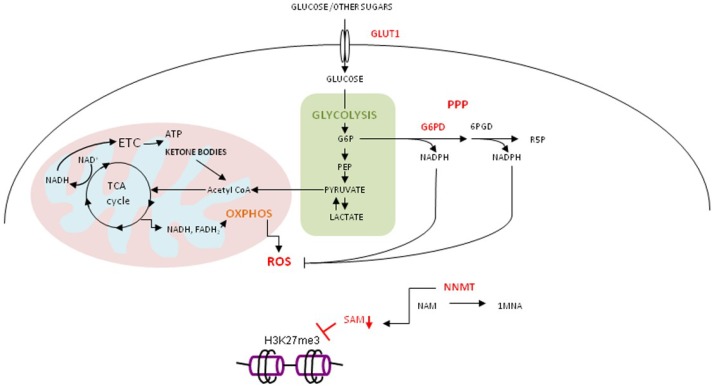
Derailed metabolism of cancer stem cell resistant to glucose deprivation. Metabolically plastic cancer cell, resistant to glucose deprivation, with high expression of GLUT1, G6PDH, G6PD and NNMT, reprograms metabolism to efficiently produce energy through utilization of sugars other than glucose (d-fructose, d-arabinose, mannan, maltotriose and dextrin), ketone bodies and lactate in OXPHOS. To counteract high level of ROS due to high OXPHOS, PPP is highly active. The upregulation of NNMT consumes methyl groups from S-adenosyl methionine (SAM) for methylation of nicotinamide (NAM). The level of SAM drops and hinders cellular methylation potential.

High level of STAT3 expression and its activity was recorded in metabolically plastic, glucose deprivation-resistant, ovarian cancer cells, accompanied by increased expression of metabolic genes *G6PD*, *GLUT1* (Glucose transporter 1) and *NNMT* (nicotinamide *N*-methyltransferase) [90]. In cancer stem cells (CSC) obtained from patients with epithelial ovarian cancer, glucose deprivation leads to enrichment of cells with a high rate of OXPHOS and PPP, joined with a high level of ROS [91]. These features represent cancer stem cell properties. High expression of GLUT1 in glucose deprivation-resistant cells allows them to utilize other sugars (d-fructose, d-arabinose, mannan, maltotriose and dextrin) and ketone bodies for energy production. Ketone bodies and lactic acid enter tricarboxylic acid (TCA) cycle to produce NADH (Nicotinamide Adenine Dinucleotide) and FADH_2_ (Flavin Adenine Dinucleotide) that feed OXPHOS to generate ATP. Elevated OXPHOS activity leads to increased levels of ROS which needs to be counterbalanced by the activity of PPP. The rate-limiting enzyme in PPP is G6PD. It generates NADPH (Nicotinamide Adenine Dinucleotide Phosphate) needed to reduce oxidized glutathione to reduced glutathione, required for reduction of ROS (Figure 1).

The role of NNMT in metabolic plasticity and cancer cell stemness is emerging. Contrary to the long-standing belief that the sole function of this enzyme is methylation of nicotinamide (NAM) and its excretion from the body, when in excess, NNMT is implicated in a plethora of important cellular processes [92]. This enzyme is overexpressed in various malignancies. In Hep G2 cells was shown that *NNMT* promoter activity depends on the activation of STAT3 [93]. The NNMT methylates NAM, creating a stable metabolic product 1-methylnicotinamide (1MNA). This reaction consumes methyl units from SAM. As a consequence, the methylation index (the ratio SAM/SAH; SAH - S-adenosylhomocysteine) of the cell changes. This way of acting puts NNMT in the central node involved in metabolic regulation of cellular methylation potential. Ulanovskaya et al. showed that 1MNA, rather than being an active, pro-tumorigenic metabolite, serves as “a stable sink” of methylation groups in cancer cells [94]. The NNMT activity has a strong, methionine concentration-dependent, impact on protein methylation. The effect on protein methylation has been observed only in the cells cultured with low methionine concentration (10-20 µM), while there was no effect in cells grown in high methionine concentration (100 µM) [94].

Likewise, the concentration of NAM varies in mammalian tissues and can impact the biological outcome of NNMT activity. In NAM-limited conditions, NNMT has a potential, in addition to negatively influencing methylation processes, to change the NAD^+^/NADH ratio. This is a consequence of the permanent loss of methylated NAM from the NAD^+^ recycling process. Another very significant observation of this study [94] is that NNMT does not equally impact methylation processes of all biomolecules. Instead, the specificity of its targeting specific methylation pathways depends most probably on the relative K_m_ values of individual methyltransferases for SAM and SAH. Methyltransferases with higher K_m_ for SAM and SAH are more sensitive to the activity of NNMT [94]. Sperber et al. [95] reported high level of NNMT in naive human pluripotent stem cells (hPSC), in which it contributed to low values of SAM and H3K27me3. Down-regulation of NNMT caused naive to primed state transition in hPSC.

Considering that curcumin also has a negative effect on STAT3 phosphorylation [96], it is not surprising that it down-regulates expression of NNMT (MDA-MB-468 breast cancer cells and HT29 colon cancer cells) [93].

### 4.3. Oxidative Stress, Curcumin and Gerometabolite NAD^+^

The oxidative stress-induced pseudohypoxic state leads to depletion of “gerometabolites”, small-molecule components of normal metabolism [97], and consequential decline in mitochondrial function [98,99]. One of the well-recognized gerometabolites is nicotinamide adenine dinucleotide, NAD^+^ [97].

NAD^+^ is an important co-factor in many metabolic reactions and is co-substrate for PARP-1 (Poly (ADP-Ribose) Polymerase 1) and SIRT1 (NAD-Dependent Deacetylase; Sirtuin-1), very important enzymes involved in cellular stress response [100]. We consider them as crucial nodes at the intersections of cellular metabolic and stress response pathways. For the cancer cell, adjustment of metabolic requirements must be precisely balanced with adequate stress response, as an imperative for survival. The pleiotropic activity of curcumin should be considered as a multilevel attack on this redundant cellular communication network in which “metabolic” meeting “inflammatory” and “oncogenic” serves as the basis for a unique cellular interactome, in accord with the postulates of Network Medicine [50].

### 4.4. Curcumin in Regulation of Metabolic and Stress Response Pathways

Curcumin supports cellular antioxidant defense through stimulating NRF2, a master regulator of ROS-scavenging enzymes. It has been shown that NRF2 may induce metabolic reprogramming by directing glucose and glutamine into the anabolic pathway. The activity of the anabolic pathway correlates with the presence of pyruvate kinase isozyme M2 (PKM2) [101]. The primary transcript of the *PKM* gene may be spliced in several different ways, and the mode of splicing is regulated by heterogeneous nuclear ribonucleoproteins (hnRNPs) [102]. The PKM1 transcript includes exon 10, while the PKM2 transcript contains part of intron 9 instead of exon 10. Of note, these two fragments are of the same length [103]. Functionally, two major protein isoforms, PKM1 (Isoform M1-PK; SwissProt Identifier P14618-2); and PKM2 (Isoform M2-PK; SwissProt Identifier P14618-1), may occur.

In contrast to the PKM1 isozyme which is normally expressed in differentiated cells, embryonic- and cancer-specific PKM2 confers a proliferative advantage to tumor cells. PKM2 can form dimers, with low activity, or active tetramers. Dimeric PKM2 diverts glucose metabolism towards aerobic glycolysis, thereby supporting biosynthetic processes, while tetrameric PKM2 promotes ATP production via OXPHOS. Since biosynthetic and energetic requirements of highly proliferative cancer cells should be simultaneously met and well adjusted, the ratio of PKM2 dimers and tetramers is critical for tumorigenesis [104]. This metabolic switch, dependent on the active axis PI3K-AKT, a part of the receptor tyrosine kinase/PI3K/AKT/mammalian target of rapamycin (RTK/PI3K/AKT/mTOR) signaling pathway, accelerates tumor proliferation and contributes to its aggressiveness [105].

**Figure 2:**
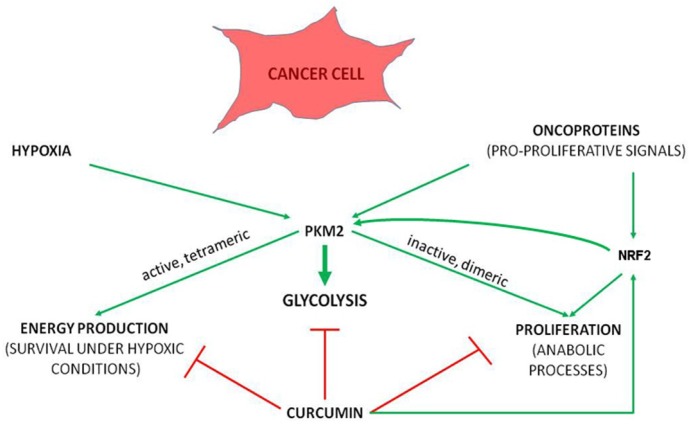
The principle underlying antitumorigenic effect of curcumin. Curcumin targets cellular processes central to the ability of cancer cell to survive by coordinately hindering aerobic glycolysis-dependent anabolic processes and energy production, despite of stimulating NRF2.

Tumor cells with constitutively activated K-ras oncogenic signaling have upregulated PKM2, which is mandatory for accumulating phosphoenolpyruvate (PEP), its shunting to an alternative glycolytic pathway and utilization for anabolic processes [106]. Despite the stimulatory effect on NRF2, curcumin antagonizes metabolic reprogramming towards glycolysis (Figure 2). One of the mechanisms involved in this process is curcumin-mediated negative regulation of TNF-α, with consequent prevention of inflammatory environment-induced onset of aerobic glycolysis. Thus, curcumin makes yet another link between inflammation and metabolism. This mechanism was shown in breast epithelial cells [107]. The same negative impact of curcumin toward glycolysis was shown in Dalton’s lymphoma, in mice [108].

The unrestricted proliferative potential of cancer cells is closely dependent on PI3K/AKT/mTOR signaling pathway [109]. For example, mTOR activation in cancer cells upregulates the expression of PKM2 through HIF-1α mediated transcriptional activation of *PKM*, and Myc-hnRNPs-mediated splicing of PKM pre-mRNA, in favor of PKM2. In a nude mouse xenograft tumor model, stable knock down of PKM2 in PC3 cells (a human prostate cancer cell line with PTEN deficiency and mTOR hyperactivation) significantly extended the survival of tumor-bearing mice [110]. Silencing of PKM2 (transduction with *shPKM2*) suppresses mTOR-mediated tumorigenesis [110]. Due to the (a) dependence of PKM2 on mTOR activation and (b) mTOR-mediated cancer cell proliferation associated with PKM2 shunting PEP into alternative glycolytic pathway supporting anabolic processes, cancer cells with hyperactive mTOR are particularly sensitive to dual inhibition of mTOR and glycolysis [111].

One of the prominent features of the multifaceted antitumorigenic effects of curcumin is its ability to simultaneously inhibit glycolysis and mTOR [112]. Recently, it has been shown that 20 µM curcumin inhibits glucose uptake and down-regulates PKM2 and lactate production in various cancer cell lines (H1299, MCF-7, HeLa and PC3) [113]. In curcumin-treated cells, decreased phosphorylation of p70S6 kinase (T389) was associated with the concomitant lowering of HIF-1α protein expression. The involvement of mTOR/HIF-1α signaling inhibition, in curcumin-mediated down-regulation of PKM2 expression, was further validated by the observation that rapamycin, a well-known mTOR inhibitor, likewise down-regulates PKM2 [113]. In addition to a negative effect on PKM2 expression, curcumin treatment, in the stated cellular models, caused decreased expression of glucose transporter GLUT1 and hexokinase II (HKII) transcripts. In HCT116 and HT29 colon cancer cells, curcumin has previously been reported to down-regulate HKII, the enzyme which catalyzes the first step in glycolysis—phosphorylation of glucose to form glucose-6-phosphate [114]. Finally, in esophageal squamous cell carcinoma EC109 cells, curcumin down-regulates the expression of glycolytic enzymes, in dose- and AMPK (AMP-Activated Kinase) -dependent manner [115].

These data indicate that the actions of curcumin are highly dependent on the context of intrinsic cellular makeup, encompassing genetic, epigenetic, metabolic and cell proliferation status, together with external influences (microenvironmental cues). All these factors may also modulate its ability for binding to various cellular proteins.

### 4.5. Metabolic Enzymes as Targets for Curcumin Binding

Approximately 60 proteins—curcumin’s binding partners, were discovered after incubating the HEI-193 human schwannoma cells with a very high concentration of biotinylated curcumin (1 mg/mL – 2.71 mM) for 30 min. The most abundant binding partners were heat shock proteins (HSPs) -70 and -90, molecular chaperones involved in the proper folding of client proteins, 3-phosphoglycerate dehydrogenase (PHDGH) and β-actin [116]. Abegg and collaborators profiled 42 proteins, covalently bound to curcumin through their cysteine residues, in HeLa cervical cancer cells [117]. A relatively small subset of Cys residues has been considered to be involved in cell signaling (contrary to ”sensing“) [118]. However, curcumin’s binding to cysteine residues of metabolic enzymes may be very important for cellular reprogramming mechanisms.

A recently published paper [119] on in situ proteomic profiling of curcumin targets in HCT116 colon cancer cell line, reports a list of 197 curcumin-binding proteins, among which are many cancer-related metabolic enzymes: GAPDH (Glyceraldehyde 3-Phosphate Dehydrogenase), PKM isozymes M1/M2, LDHA (L-Lactate Dehydrogenase A), MDH1/2 (cytoplasmic/mitochondrial Malate Dehydrogenase), SHMT2 (Serine Hydroxymethyltransferase; mitochondrial), ADP/ATP translocase 1. Additionally, curcumin targets PARP-1, NAD^+^ -consuming enzyme involved in numerous cellular processes including DNA-damage detection and repair, transcription and intracellular localization and activity of many proteins. Binding to numerous hnRNPs, including the alternative splicing repressors hnRNP A1/A2 which influence PKM splicing [103], point to the possible role of curcumin in regulating alternative splicing. So far, its influence on splicing was shown in fibroblasts from patients with SMA type II (Survival of Motor Neuron 2) in which curcumin increased the proportion of *SMN2*_exon 7-containing transcript [120].

How curcumin binding to its protein targets influences their activity is mostly unknown. The SiteMap calculation performed by Angelo et al. [116] predicted the binding of curcumin to NAD^+^ binding site on PHGDH, which is crucial for PHGDH enzymatic activity. The curcumin binding enzymes, PHGDH and SHMT2, belong to the serine-glycine metabolic pathway. Both amino acids, serine and glycine, serve as intermediates for the biosynthesis of other amino acids, nucleic acids and lipids. What is the possible scenario of curcumin’s binding to these enzymes?

#### Phosphoglycerate Dehydrogenase and Serine Hydroxymethyltransferase 2

PHGDH, the first, rate-limiting enzyme in the pathway of serine biosynthesis, catalyzes the transition of 3-phosphoglycerate (3PG) into 3-phosphohydroxypyruvate, using NAD^+^/NADH as a cofactor. Shunting of the glycolytic intermediate 3PG to serine synthesis is permitted by PKM2. Low activity of PKM2 in tumor cells leads to an accumulation of its substrate PEP, which participates in phosphorylation and the catalytic activation of phosphoglycerate mutase (PGAM1) [106]. As a consequence, there is an increase of its product, 2-phosphoglycerate, which activates PHGDH and serine biosynthesis [121].

PHGDH protein level is elevated in 70% of estrogen receptor (ER)-negative breast cancers and suppression of PHGDH in breast cancer cell lines decreases cell proliferation and reduces serine synthesis [122]. In addition to increasing PHGDH, an increase of nicotinamide phosphoribosyltransferase (NAMPT), a rate-limiting enzyme of NAD^+^ salvage pathway, was also shown in ER-negative breast cancer lines and patient-derived breast tumors [123]. Their interconnection was shown experimentally on PHGDH^high^MDA-MB-468 and PHGDH^low^ MDA-MB-231 breast cancer cells by treating them with well-established NAMPT inhibitor (FK866). This treatment caused a drop in NAD^+^ level and almost completely abrogated serine synthesis in PHGDH^high^MDA-MB-468 cells, while the drop in NAD^+^ level and serine synthesis was insignificant in PHGDH^low^ MDA-MB-231 cells [123].

Although pursued in cancer treatment, NAD^+^ salvage pathway inhibitors exert various efficacy. Thus, it was suggested that NAMPT inhibitors may be effective for treating a subset of PHGDH-dependent cancers [123]. Therefore, it is possible that curcumin, in addition to inhibiting PHGDH directly by binding to its NAD^+^ pocket [116], hinders its activity indirectly by down-regulating expression of NAMPT (also known as visfatin) as has been demonstrated in breast cancer cell lines (MDA-MB-231, MDA-MB-468, and MCF-7) [124]. Knocking down PHGDH in HeLa cells significantly inhibited cell proliferation and increased cisplatin chemosensitivity [125].

Serine can be directly converted to glycine by two serine hydroxymethyltransferases: cytoplasmic (SHMT1) and mitochondrial (SHMT2). SHMT2 is the main source of glycine in proliferating cells [126] and its importance for tumor cells survival in hypoxia was demonstrated in glioblastoma multiforme (GM) [127].

**Figure 3:**
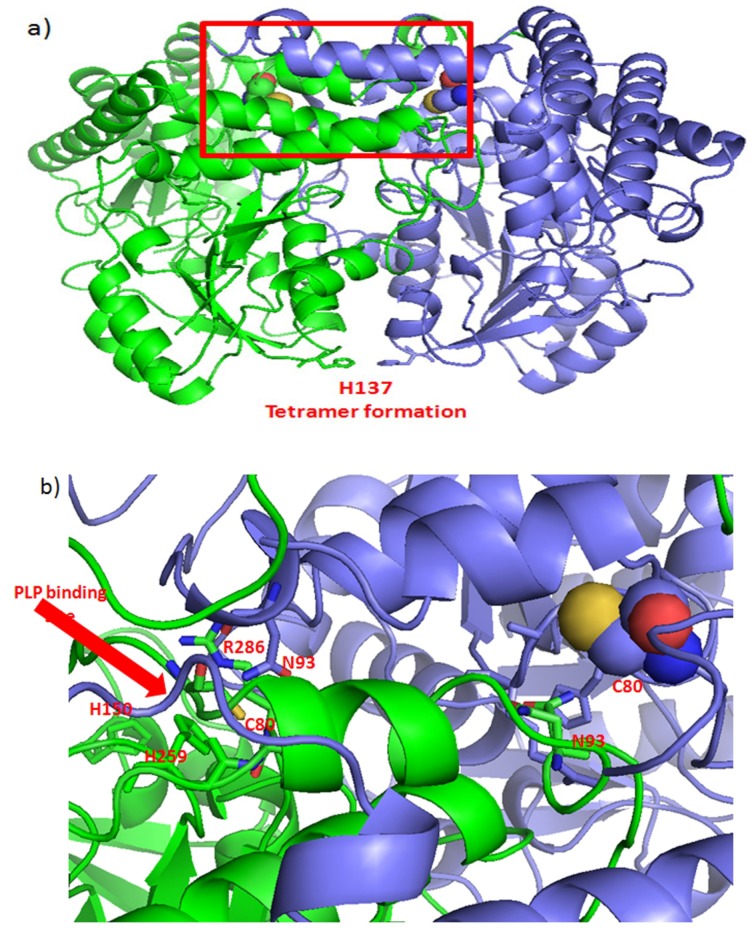
Possible effects of curcumin’s binding to SHMT2 Cys80. (**a**) The symmetric dimer of SHMT2 (PDB: 4PVF) showing amino acid residues Cys80 (spheres) and His137 (sticks) participated in the tetramer formation [128]. Monomers are shown in green and purple. (**b**) The part of the 4PVF structure within red rectangular in (**a**), with marked amino acid residues making interactions with the nonmodified Cys80. The pyridoxal 5’-phosphate (PLP) binding site is encompassed by marked His150 and His259.

Competent eukaryotic SHMT2 is a tetrameric protein built as dimers of “tight” dimers which correspond to its minimal catalytic active units. A dimer-to-tetramer transition is triggered by the binding of the isozyme SHMT2 cofactor, pyridoxal 5’-phosphate (PLP) [128]. Curcumin-binding Cys80 [116] indirectly influences the formation of the binding site for PLP, through interacting with residues Arg283 and Gly284 to Arg286 (Figure 3). Cys80 also participates in protein-protein interaction of the tight dimer, through interacting with Asn93 of the other chain. Thus, covalent binding of curcumin to Cys80 may be expected to impact both, the structure and the catalytic activity of the active form of SHMT2.

As recently described in GM, SHMT2 activity limits the activity of PKM2 and reduces oxygen consumption, thereby eliciting a metabolic state permissive of tumor cells survival in poorly vascularized tumor regions. It is plausible to expect that curcumin’s binding to Cys80 may hinder formation of SHMT2 tetramers, inhibit SHMT2 activity and prevent PKM2-mediated metabolic reprogramming, needed for facilitating cell survival in hypoxia [127]. The negative effect of elevated SHMT2 activity on PKM2 may stem from the increased catabolism of serine, a known PKM2 activator [129]. Transformation of serine in the mitochondria by SHMT2 increases the cellular NADPH/NADP^+^ ratio and decreases ROS production [130]. Consistent with its pro-survival role under hypoxia and its role in limiting ROS, SHMT2 was recently identified as a potential cancer driver gene [131].

## 5. Potential for Therapeutic Application?

The amplification of the chromosomal locus (1p12) containing the *PHGDH* gene, has been reported in melanomas and triple-negative breast cancers [132]. The enzyme has been recognized as a potential therapeutic target for NAD-competitive inhibitors in PHGDH-amplified breast cancer [133]. In renal carcinoma, in which PHGDH represents the key regulator of the HIF-2α pathway, targeted inactivation of PHGDH may be promising for treating patients resistant to HIF-2α antagonists [134].

When considering PHGDH prognostic significance in a broad context, bioinformatic analysis of human breast and lung cancer mRNA data sets found high *PHGDH* expression to be a negative prognostic marker in breast cancer patients in seven out of 17 breast cancer datasets [135]. However, the increased expression of the PHGDH mRNA did not appear to have any prognostic value in seven analyzed lung cancer datasets, highlighting the importance of the cell-specific molecular background as the key determinant for activating specific signaling pathways. The level of their dependency on specific molecules/enzymes should be put in the context of redundant signaling mediators, the presence of which is, again, cell-type specific.

The fact that high expression of *SHMT2* in 10 out of 17 breast cancer datasets predicted negative prognosis, seems to be in accord with this presumption. Four breast cancer datasets shared both enzymes, PHGDH and SHMT2, as negative prognostic factors. Finally, NRF2 has an important role in transcriptional regulation of genes coding for these enzymes, in ATF4-dependent fashion. As shown in the model of non-small cell lung cancer (NSCLC), high expression of NRF2, joined with high expression of both PHGDH and SHMT2, represents the clinical marker of the poor outcome [136].

Increased SHMT2 positively correlates with the breast cancer grade [137] and is associated with worse relapse-free survival (RFS), distant metastases, and overall survival (OS) in breast cancer patients (*N* = 801) [131]. Similarly, high SHMT2 expression in hepatocellular carcinoma (HCC) significantly correlates with decreased OS, lymph node metastases and HCC grade [138]. For exerting its activity, the SHMT2 must interact with sirtuin SIRT5 which desuccinylates SHMT2, at lysine 280. This modification seems to be critical for SHMT2 active involvement in tumor growth [139]. This is a good example of the complexity in the field and, as already mentioned, is necessary for the multilevel ways of research that need to include as many explorations as possible of various interactions.

## 6. Conclusions

The complex hierarchy in regulating inflamm-aging related pathological network is still incompletely understood, notwithstanding ever-increasing knowledge regarding every part of the process.

A recent report showing that curcumin acts as a prodrug, while its oxidative metabolites may bind to various cellular proteins, is of a great importance for understanding curcumin’s action in various pathological conditions/diseases. Discovery of various proteins to which curcumin can bind adds an additional challenge for understanding all modalities resulting from its pleiotropic actions. In this review, we have dedicated a great deal of attention to cancer, which represents a disease of derailed metabolic and signaling pathways which are tightly interwoven. We have yet to discover molecules or processes which supervise and are indispensable for the establishment of an oncometabolic network comprising malignantly transformed and surrounding „normal“ cells. With respect to an approach to therapy, personalized medicine has made great progress. However, developing an integrated approach, in which personalized medicine is applied as much as possible, will present a very demanding task for the future. Taking into account that the most prominent feature of cancer cells - uncontrolled division - relies on numerous, mutually and closely inter-dependent pathways, the critical nodes in this pro-proliferative, oncometabolic network must be identified and combated, according to proposed rules postulated by Network Medicine.

Since these crucial regulatory nodes are diverse and exist at several levels, the introduction of a pleiotropic molecule, as a part of the therapeutic effort, would make sense. Such a pleiotropic molecule should be able to selectively maintain the homeostatic network in normal cells, while attacking cancer cells through affecting the „unbalanced“ state to which they are addicted. This is a very demanding task, asking for much knowledge and dedication to the challenge posed.

Curcumin may have features needed to help meet this challenge. In this review, we have tried to present the most current data from a perspective that would enable establishing connections and functional links between the specifics of inflamm-aging and the cancer cell’s metabolism, its proliferative potential, and curcumin’s pleiotropic activity. We believe that it opens the door to a wide range of therapeutic opportunities for targeting cell-type specific nodes as crucial points for targeting a functional oncometabolic signaling network, while recognizing that other aspects need to be studied.

## Abbreviations

1MNA	1-Methylnicotinamide
3-PG	3-Phosphoglycerate
AD	Alzheimer’s Disease
AMPK	AMP-Activated Kinase
ATP	Adenosine Triphosphate
CBR1	Carbonyl Reductase 1
CRP	C-Reactive Protein
CS	Cellular Senescence
CSC	Cancer Stem Cells
ER	Estrogen Receptor
FADH_2_	Flavin Adenine Dinucleotide
G6PD	Glucose-6-Phosphate Dehydrogenase
GAPDH	Glyceraldehyde 3-Phosphate Dehydrogenase
GM	Glioblastoma Multiforme
GLUT 1	Glucose Transporter 1
GSH	Glutathione
GSTP1	Glutathione-S-Transferase Phi 1
HCC	Hepatocellular Carcinoma
HIF-1α	Hypoxia-Inducible Factor 1α
HKII	Hexokinase II
HNC	Head and Neck Carcinoma
IKK b	Inhibitor of Nuclear Factor Kappa-B Kinase Subunit Beta
IL-17R	IL-17 Receptor
LDHA	L-Lactate Dehydrogenase A
MBN	MidBrain Neuron
MCP-1	Monocyte Chemoattractant Protein-1
MDH	Malate Dehydrogenase
MMP-2	Matrix Metalloproteinase 2
NADH/NAD^+^	Nicotinamide Adenine Dinucleotide
NADPH/NADP^+^	Nicotinamide Adenine Dinucleotide Phosphate
NAM	Nicotinamide
NAMPT	Nicotinamide Phosphoribosyltransferase
NF-κB	Nuclear Factor Kappa-Light-Chain-Enhancer of Activated B Cells
NNMT	Nicotinamide *N*-Methyltransferase
NOROS	Nitric Oxide and Reactive Oxygen Species
NQO-1	NAD(P)H Quinone Dehydrogenase 1
NRF2	Nuclear Factor (Erythroid-Derived 2)-Like 2
OS	Overall Survival
OXPHOS	Oxidative Phosphorylation
PARP-1	Poly (ADP-Ribose) Polymerase 1
PD	Parkinson’s Disease
PEP	Phosphoenolpyruvate
PGAM1	Phosphoglycerate Mutase
PKM	Pyruvate Kinase
PPP	Pentose Phosphate Pathway
PR	Progesterone Receptor
R5P	Ribulose 5-Phosphate
RFS	Relapse Free Survival
ROS	Reactive Oxygen Species
SAH	S-Adenosylhomocysteine
SAM	S-Adenosylmethionine
SEMF	Subepithelial Myofibroblast
SHMT2	Serine Hidroxymethyltransferase 2
STAT3	Signal Transducer and Activator of Transcription 3
TCA	Tricarboxylic Acid
TGF-b	Transforming Growth Factor Beta
TNF-a	Tumor Necrosis Factor-Alpha
VEGF	Vascular Endothelial Growth Factor
